# High-risk extracorporeal membrane oxygenation in immunocompromised children with acute respiratory failure: a retrospective cohort study

**DOI:** 10.3389/fonc.2025.1613864

**Published:** 2025-07-08

**Authors:** Liudmila Belevskaia, Florian von Borell, Ulrich Baumann, Rita Beier, Harald Köditz

**Affiliations:** ^1^ Department of Pediatric Cardiology and Intensive Care Medicine, Hannover Medical School, Hannover, Germany; ^2^ Department of Paediatric Pulmonology and Neonatalogy, Hannover Medical School, Hannover, Germany; ^3^ Department of Pediatric Hematology and Oncology, Hannover Medical School, Hannover, Germany

**Keywords:** ECMO, immunosuppression, inborn immunodeficiancy, cancer, respiratory failure, HCT (hematopoietic cell transplantation), pediatric

## Abstract

**Background:**

Extracorporeal membrane oxygenation (ECMO) is increasingly being utilized in pediatric patients with severe respiratory failure, extending its use to high-risk patients, including those who are immunocompromised. Despite its growing application, reports on outcomes and prognostic factors in this specific population are scarce, highlighting a gap in our understanding.

**Methods:**

This retrospective cohort study analyzed the outcomes of 19 immunocompromised pediatric patients who received ECMO for respiratory failure at our institution between 2006 and 2023. Patients were classified as immunocompromised due to conditions such as cancer, hematopoietic cell transplantation (HCT), primary immunodeficiency or receiving immunosuppression for a chronic (auto-) inflammatory disease. Data on patient demographics, baseline laboratory and ventilation parameters were collected and compared between survivors and non-survivors.

**Results:**

The median age of patients was 12.1 years, and the majority suffered from infectious pneumonia leading to respiratory failure. The median duration of ventilation before ECMO was 5 days, and ECMO support lasted a median of 19 days. The hospital mortality rate in this cohort was 74% (14/19). All patients who had undergone HCT or a primary immunodeficiency did not survive. Non-survivors exhibited significantly higher median C-reactive protein levels and more bleeding complications. Other laboratory and respiratory parameters, as well as vasopressor requirements, pSOFA, and P-PREP scores, were similar across survivors and non-survivors.

**Conclusion:**

The treatment of immunocompromised pediatric patients with ECMO for respiratory failure presents notable challenges. This study highlights the complexity of predicting outcomes in this group, as traditional laboratory and respiratory parameters were not distinctly different between survivors and non-survivors. These findings indicate a need for continued research and nuanced clinical approaches to improve care and outcomes in this vulnerable population.

## 1 Introduction

Extracorporeal membrane oxygenation (ECMO) is well recognized as a valuable therapeutic option for children with severe respiratory failure, particularly when mechanical ventilation fails to maintain essential gas exchange (1, 2). This is evidenced by an increase in ECMO utilization over the past two decades and can be attributed to technological advancements and achievements in critical care (3). Alongside these developments, the indications for ECMO have expanded, increasingly encompassing higher-risk groups, including historic contraindications like immunosuppression (4–6).

A compromised immune system is a well-recognized risk factor associated with higher mortality in patients receiving ECMO support (7–9). Notably, the reported survival rate for immunocompromised children on ECMO therapy ranges between 30-40%, which is substantially lower than the reported 69% overall survival rate for children receiving respiratory ECMO support in recent years (7, 10–14).

The prognosis for children receiving ECMO following hematopoietic cell transplantation (HCT) tends to be particularly poor, underscoring the imperative for cautious and thoughtful application of ECMO in this patient subgroup (6, 15). Recent trends indicate an improvement in the prognosis for immunocompromised pediatric patients needing ECMO support (16). This has contributed to a growing acceptance of ECMO in situations where the treatment trajectory appears more favorable (17, 18).

The decision to initiate ECMO for respiratory failure in immunocompromised patients is complex and must be individualized, considering the prognosis of the underlying disease and the severity of acute complications leading to respiratory failure (18–20). It necessitates a nuanced understanding of the disease, its prognosis, and the patient’s individual risk profile to optimize patient selection.

Our study aims to contribute to this evolving field by characterizing the outcomes of immunocompromised children who received ECMO therapy for respiratory failure at our institution. Through this analysis, we seek to enhance the understanding of this unique patient population and provide a stronger foundation for future patient selection and management strategies.

## 2 Material and methods

For this retrospective cohort single center study, we included all patients younger than 18 years of age who were admitted to the pediatric intensive care unit (PICU) of the Hannover Medical School with a compromised immune status and who received ECMO for respiratory failure. We included all admissions between 2006 and October 2023. We defined patients as immunocompromised if one of the following entities was present at ECMO initiation: I) immunosuppressive treatment after HCT; II) received chemotherapy for hematological malignancy or solid tumor prior to ECMO initiation and/or neutropenia (<500/µl); III) immunosuppressive treatment for > 30 days for an autoimmune disease, solid organ transplant or other inflammatory diseases IV) primary immunodeficiency (PID). To be considered as immunosuppression corticosteroid doses had to exceed 0.5 mg/kg/d for more than 30 days directly preceding ECMO initiation (21). For the purposes of this study, chemotherapy refers to the administration of cytotoxic, antineoplastic agents for cancer-directed treatment.

Patients would be allocated to ECMO following institutional regulations based on the Extracorporeal Life Support Organization (ELSO) guideline for pediatric respiratory failure (1). Patients requiring ECMO for respiratory failure were cannulated in a veno-venous (VV) configuration, veno-arterial (VA)-ECMO was applied only in cases of additional cardiac failure or when patient anatomy would not allow for VV-cannulation. Cannulation sites were jugular and/or femoral due to individual patient anatomy and vessel size. Weaning from ECMO was performed in accordance with the ELSO guidelines (4).

The primary outcome was survival to hospital discharge, but data including 6-month survival as well as baseline parameters prior to ECMO initiation and ECMO-related complications will also be reported.

All patient-related data was derived from the electronic patient data management system used by the PICU. Recorded data included age, weight, diagnosis, reason for respiratory failure, type of immunosuppression, treatment specifications. Furthermore, ECMO mode, runtimes and complications as defined by ELSO (22), respiratory parameters, and laboratory data were recorded. The vasoactive-inotropic score (VIS) was calculated to compare vasoactive medication between patients (23). The time point immediately before the start of ECMO treatment was referred to as the baseline. Baseline data were recorded for the hour immediately before ECMO cannulation. The *pediatric Sequential Organ Failure Assessment score* (pSOFA) as well as the *Pediatric Pulmonary Rescue with Extracorporeal Membrane Oxygenation Prediction score* (P-PREP) were calculated at baseline to estimate severity of illness and to predict mortality after ECMO initiation (24, 25).

Data were summarized using frequencies and percentages for categorical variables and median and interquartile range (IQR) for continuous variables. Data were compared between survivors and non-survivors using Fisher’s exact test for dichotomous variables and the Wilcoxon rank sum test for continuous variables. Differences were considered statistically significant when p value was less than 0.05. Statistical tests were performed using R software version 4.0.3, “Bunny-Wunnies Freak Out”, copyright 2020, The R Foundation for Statistical Computing.

The study complied with the Declaration of Helsinki, ethical approval for this study was waived by the ethics committee of Hannover Medical School.

## 3 Results

Between 2006 and 2023 we identified 19 patients who received ECMO for respiratory failure and were categorized as high-risk patients being immunocompromised. The median age at admission was 12.1 years, ranging from 5 month to 17 years of age. A total of 9 patients had a history of malignant disease; 5 were receiving chemotherapy at ECMO initiation. Of the remaining oncology patients, 3 had undergone HCT, and one had monomorphic PTLD after liver transplantation. In total, 4 patients received HCT, including one with thalassemia. Rituximab was administered in 3 patients: the one with PTLD, one for kidney transplant rejection, and one for induction therapy in granulomatosis with polyangiitis. Long-term corticosteroid use was present in 3 patients (including one post-lung transplant), and 4 patients had PID (**Table 1**). The majority of patients (15/19) suffered from infectious pneumonia with identified pathogens causing respiratory failure.

**Table 1:** Patient characteristics.

Pt	Diagnosis	Group IS	Details on IS prior to ECMO	Causes for respiratory failure	ECMO-run (days)	Weaned from ECMO	Hospital survival
*1*	T-ALL	HCT	allogenic, 8 days prior to ECMO	*CMV* pneumonia	9^vv^	no	no
*2*	AML, M5	HCT	allogenic, 134 days prior to ECMO	*Pneumocystis jirovecii* pneumonia, *S. epidermidis* and *S. hominis* sepsis	30^vv^	no	no
*3*	Medulloblastoma °IV	HCT	autologous, 84 days prior to ECMO	Chemotherapy associated alveolar wall damage	7^vv^	no	no
*4*	Homozygous beta thalassemia	HCT	allogenic, 45 days prior to ECMO	*CMV* pneumonia	7^vv^	no	no
*5*	AML, M4Eo.	ONC	week 2 of induction*, ANC 0/µl	Diffuse alveolar hemorrhage	8^vv^	no	no
*6*	common ALL	ONC	week 5 of induction*, ANC 85/µl	*S. mitis* sepsis, pneumonia	11^vv^	yes	yes
*7*	T-ALL, tumor lysis syndrome	ONC	week 1 of induction*, ANC 5530/µl	Tumor mass obstruction of left main bronchus, cardiac failure due to tumor lysis syndrome	5^va^	yes	yes
*8*	common ALL	ONC	week 6 of consolidation*, ANC 800/µl	*Influenza* (H1N1) pneumonia	38^vv^	yes	yes
*9*	pre-B-ALL	ONC	week 3 of re-induction*, ANC 0/µl	*HMPV* pneumonia	19^vv^	no	no
*10*	Cystic fibrosis, LuTX	CS	long-term + 5-day 20mg/kg/d MP pulse	*Candida albicans* sepsis, pneumonia	23^vv^	yes	no
*11*	Colitis ulcerosa, backwash ileitis	CS	>30 days of PS 1.1mg/kg/d	*Candida albicans* pneumonia with *S. aureus* superinfection, hemorrhagic infarction	22^vv^	no	no
*12*	Juvenile nasopharyngeal angiofibroma	CS	>30 days 0.5-07mg/kg/d + MP pulse	Pneumonia without identified pathogen	30^vv/va^	yes	no
*13*	Granulomatosis with polyangiitis	RTX	remission-induction, 375mg/m^2^/week for 4 weeks	Influenza (H1N1) pneumonia	11^vv^	yes	yes
*14*	Monomorphic PTLD following LTX for biliary atresia	RTX	PTLD treatment 375mg/m^2^/week for 4 weeks	*Pneumocystis jirovecii* pneumonia	20^va^	yes	yes
*15*	Jeune syndrome, kidney transplantation and rejection	RTX	375mg/m^2^/week for 4 weeks for treatment of rejection	SARS-CoV-2 pneumonia	20^vv^	yes	no
*16*	SCID	PID	–	RSV pneumonia	8^vv^	no	no
*17*	Mannose-binding lectin (MBL) deficiency (homozygous BB-mutation), RPGN	PID**	–	Pulmonary aspergillosis (*Aspergillus fumigatus*)	45^vv^	no	no
*18*	Hypogamma-globulinemia, impaired T-cell proliferation of suspected genetic etiology	PID	–	HSV pneumonia	38^vv/va^	yes	no
*19*	Chronic granulomatous disease	PID	–	Pulmonary aspergillosis (*Aspergillus fumigatus*)	1^vv^	no	no

5-ASA, 5-aminosalicylic acid; ANC, absolute neutrophil count; CMV, Cytomegalovirus; CS, corticosteroids; HMPV, Human metapneumovirus; IS, immunosuppression; LTX, liver transplantation; LuTX, lung transplantation; MBL, Mannose-binding lectin; MMF, Mycophenolate mofetil; MP, methylprednisolone; ONC, oncological; PID, primary immunodeficiency; PS, prednisolone; Pt, patient; PTLD, post-transplant lymphoproliferative disorder; RTX, rituximab; RPGN, rapidly progressive glomerulonephritis; SCID, severe combined immunodeficiency; ^va^ VA-ECMO; ^vv^ VV-ECMO; ^vv/va^ conversion VV- to VA-ECMO; *time point: beginning of the respective chemotherapy phase preceding ECMO initiation; **repeatedly very low measured values of serum MBL during infection (serum MBL: 67ng/ml, reference (no ongoing infection) > 450ng/ml).

**Figure 1:**
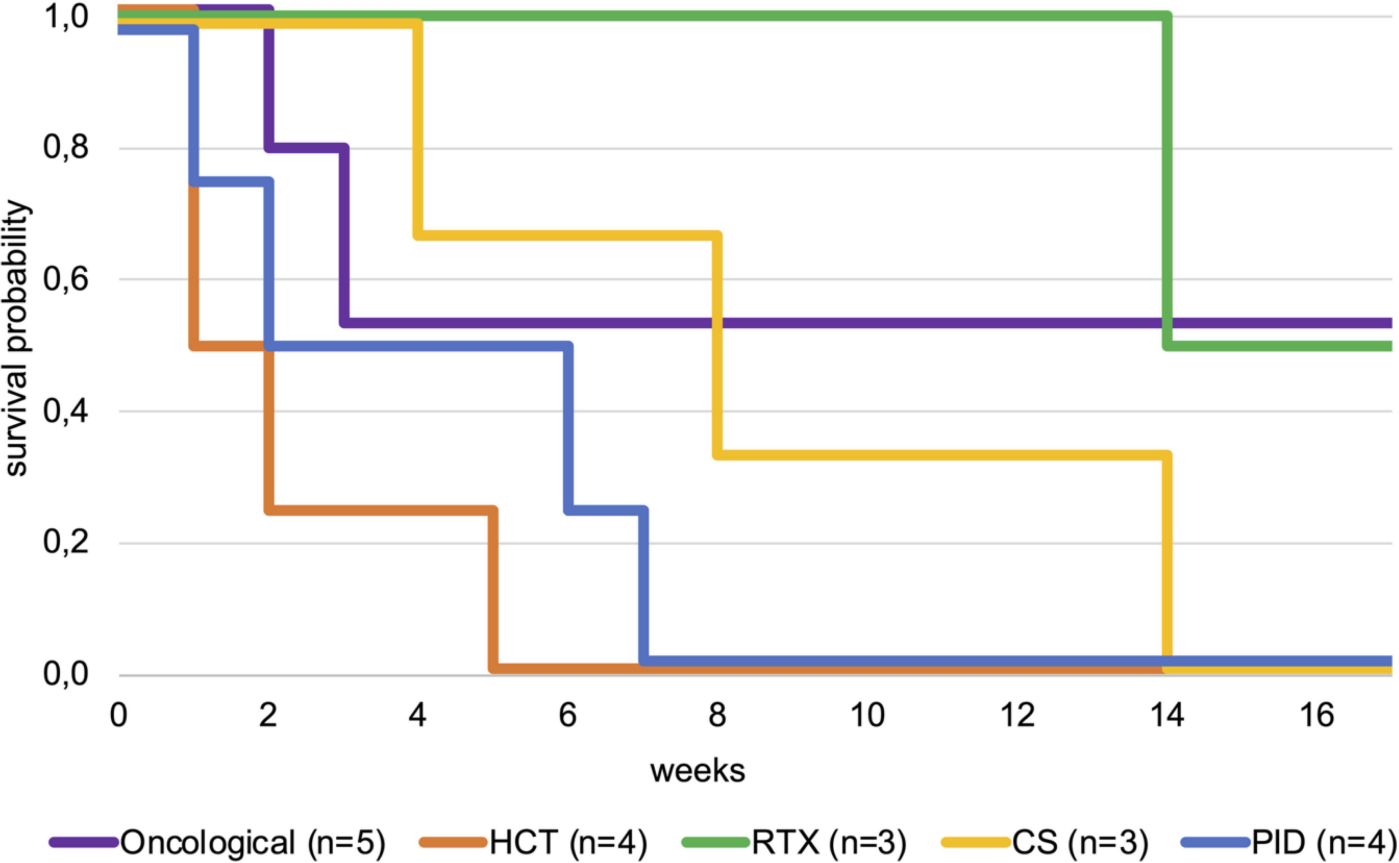
Kaplan-Meier plot comparing survival probability until discharge for patients after HCT, oncological patients (without history of HCT), patients receiving rituximab or corticosteroids and patients with primary immunodeficiency. CS, corticosteroids; HCT, hematopoietic cell transplantation; PID, primary immunodeficiency; RTX, rituximab.

Initial cannulation strategy was veno-arterial (VA) in 2 patients and veno-venous (VV) in 17 patients of whom 2 got converted to VA during the ECMO-run. Initial VA-ECMO was chosen for one patient due to combined cardiac and respiratory failure and for another due to anatomical barriers for VV cannulation. All patients were intubated prior to commencement of ECMO and had a median ventilation time of 5 days (IQR: 1–11 days) before cannulation. The median duration of ECMO support was 19 days (IQR: 8–27 days). In total, 10/19 (53%) patients could not be weaned from ECMO, and 14/19 (74%) patients did not survive the hospital stay. The median length of PICU stay was 26 days (IQR: 12–46 days). All 5 patients who survived to hospital discharge were still alive 6 months later. All 4 patients after HCT failed to wean from ECMO. Of the 4 patients with primary immunodeficiency only 1 was successfully weaned from ECMO but deceased before discharge from hospital. All patients are characterized in **Table 1**, and survival probability within the subgroups is displayed in **Figure 1**.

When comparing survivors at hospital discharge and non-survivors (**Table 2**), median C-reactive protein (CRP) was significantly higher in the non-survivor group (57 mg/L vs. 198 mg/L; p = 0.03). Regarding other baseline laboratory parameters, there were no significant differences in white blood cell count, hemoglobin levels, platelet count, creatinine, pH or lactate levels between the groups. Baseline respiratory parameters like positive end-expiratory pressure (PEEP), peak inspiratory pressure (PIP), mean airway pressure (Pmean), Oxygenation Index (OI), carbon dioxide partial pressure (pCO2), arterial oxygen partial pressure (PaO2), and ventilation duration prior to ECMO were similar across both groups. Moreover, neither vasopressor requirements expressed by VIS score nor the pSOFA or P-PREP score differed significantly. Neither the duration of ECMO support nor the length of PICU stay differed significantly between survivors and non-survivors, with the latter being 31 vs. 21.5 days, respectively (p = 0.49). Non-survivors had more bleeding complications (20% vs. 79%; p = 0.04) and a higher total number of complications (1 vs. 3.5, p = 0.048) during ECMO support. There was no significant difference in age or weight.

**Table 2:** Comparing patient data of survivors at hospital discharge and deceased.

Variables	Total	Hospital survivors	Deceased	p-value
n	19	5	14	
Age (years)	12.1 (3.4, 16)	10.1 (3.5, 13.4)	12.2 (5, 16.2)	0.64
Weight (kg)	34 (18, 54.5)	27 (21, 33.6)	41 (17.3, 55)	0.31
High-risk profile				
Oncological (n)	5	3	2	
HCT (n)	4	0	4	
CS-therapy (n)	3	0	3	
Rituximab (n)	3	2	1	
PID (n)	4	0	4	
pre ECMO baseline data				
Ventilation before ECMO, days	5 (1, 10)	7 (2, 13)	4.5 (0.25, 10)	0.40
PEEP (cm H_2_O)	15 (12, 16)	16 (14, 16)	14.5 (12, 15.8)	0.30
PIP (cm H_2_O)	35 (32, 38)	33 (32, 36)	35.5 (32, 39.5)	0.40
Pmean (cm H_2_O)	23 (20, 25)	20 (20, 23)	23.5 (18, 25)	0.78
pCO2 mmHg	62 (50, 75.5)	54 (54, 60)	69.5 (50, 79.3)	0.23
OI	35.7 (26.3, 42.7)	33.7 (29, 36.4)	37.6 (26, 43)	1.0
VIS	8 (2, 12.5)	5 (0, 8)	10 (3.3, 12.8)	0.38
Creatinine µmol/L	50 (30, 108.5)	29 (17, 70)	69.5 (42, 112)	0.23
CRRT	37%	40%	36%	1.0
WBC/nl	9.3 (2.8, 13.5)	9.3 (2.6, 11.8)	9.7 (3.1, 15.5)	0.78
Platelets/nl	105 (43, 202)	86 (48, 110)	141.5 (47, 241)	0.58
CRP mg/L	150 (48.5, 259)	57 (23, 87)	198 (113, 318)	**0.03**
Lactate mmol/L	1.9 (1, 2.7)	1.9 (1.5, 2.3)	1.8 (1, 2.8)	0.68
pSOFA	13 (11.5, 16)	14 (13, 15)	12 (11.3, 16)	0.82
P-PREP	22 (13, 29.5)	18 (14, 22)	24.5 (11.3, 31)	0.64
ECMO data				
Runtime, days	19 (8, 26.5)	11 (11, 20)	19.5 (8, 28.3)	0.85
ECMO complications				
Mechanical	58%	40%	64%	0.63
Hemorrhagic	63%	20%	79%	**0.04**
Neurologic	11%	0%	14%	1.0
Renal	37%	20%	43%	0.6
Cardiovascular	21%	20%	21%	1.0
Pulmonary	26%	0%	36%	0.26
Infection	21%	20%	21%	1.0
Metabolic	37%	20%	43%	0.60
Nr of complications	2 (1, 3.5)	1 (1, 2)	3.5 (2, 4)	**0.048**

Values are reported as median (Q_1_, Q_3_) OR percentage. CS, corticosteroids; CRRT, continuous renal replacement therapy; HCT, hematopoietic cell transplantation; OI, Oxygenation Index; PEEP, positive end expiratory pressure; PID, primary immunodeficiency; PIP, positive inspiratory pressure; Pmean, mean airway pressure; VIS, vasoactive-inotropic score; WBC, white blood cell count.

Bold values mean statistically significant.

Furthermore, no significant differences in baseline data, including CRP, were observed between the 9 patients who were successfully weaned from ECMO and those who were not (**Table 3**).

**Table 3:** Comparing patients who were successfully weaned from ECMO to patients who died on ECMO.

Variables	Weaned, n=9 median (IQR)	Died, n=10 median (IQR)	p-value
Age (years)	11.8 (10.2)	12.2 (11.7)	0.74
Weight (kg)	33.6 (33)	41 (36.3)	0.87
Intubation before ECMO, days	4 (6)	6 (10)	0.77
OI	33.7 (7.4)	40.8 (17)	0.87
VIS	5 (8)	11.5 (12.6)	0.35
Creatinine µmol/L	50 (41)	93 (78.3)	0.46
WBC/nl	9.3 (7.7)	9.7 (19)	0.74
Platelets/nl	110 (133)	99.5 (163.5)	0.46
CRP mg/L	87 (156)	193.5 (207.8)	0.17
Lactate mmol/L	1.5 (1.4)	2 (1.7)	0.44
pSOFA	13 (5)	14 (4.8)	0.15

OI, Oxygenation Index; VIS, Vasoactive-Inotropic Score; WBC, white blood cell count.

As displayed in **Table 4**, all patients who survived until discharge lived and were thriving 6 months later. The causes of death for the 14 deceased patients are summarized in **Table 5**.

**Table 4:** Survivors’ outcomes at 6 months after discharge.

Pt	Diagnosis	Outcome 6 month after discharge
*6*	common ALL	in complete remission, thriving
*7*	T-ALL	in complete remission, thriving, secondary sclerosing cholangitis, mild cognitive developmental delay, autism spectrum disorder
*8*	common ALL	in complete remission, thriving
*13*	Granulomatosis with polyangiitis	in complete remission, thriving, CKD G2A2 (KDIGO), bilateral hearing impairment
*14*	Biliary gallbladder atresia, Liver transplantation, PTLD	thriving, resolved episode of mild acute transplant rejection (RAI 3)

CKD, chronic kidney disease; KDIGO, Kidney Disease Improving Global Outcomes – staging criteria; Pt, patient; PTLD, post-transplant lymphoproliferative disorder; RAI, rejection activity index.

**Table 5:** Causes of death.

Pt	Weaned from ECMO	Cause of death
*1*	no	MODS
*2*	no	MODS
*3*	no	irreversible lung damage – withdrawal of ECMO support
*4*	no	MODS, uncontrolled infection
*5*	no	MODS
*9*	no	MODS, uncontrolled infection
*10*	yes	deterioration of uncontrolled infection after ECMO weaning progression to MODS and death 30 days after ECMO
*11*	no	MODS
*12*	yes	after ECMO weaning recurrent severe respiratory failure and MODS; decision against re-initiation of ECMO and death 65 days after ECMO
*15*	yes	partial respiratory improvement led to ECMO weaning, prolonged PICU course without recovery of other organ systems, death due to sepsis and MODS 73 days after ECMO
*16*	no	no respiratory improvement, MODS, withdrawal of ECMO therapy
*17*	no	inadequate respiratory recovery due to uncontrolled aspergillus pneumonia, withdrawal of ECMO therapy
*18*	yes	recurrent respiratory deterioration, uncontrolled HSV infection, no re-initiation of ECMO, death 1 day after ECMO therapy
*19*	no	uncontrolled mediastinal bleeding

MODS, multiple organ dysfunction syndrome; Pt, patient.

## 4 Discussion

ECMO is a recognized and recommended option for children experiencing acute respiratory failure and inadequate gas exchange while maintaining lung protective ventilation (2, 26). The indications and scenarios for ECMO support are expanding, and being immunocompromised is no longer considered a clear contraindication in pediatric respiratory failure (1). However, it has been demonstrated that a compromised immune system independently poses a high-risk factor for a worse prognosis and higher mortality during ECMO treatment (1, 8, 14). In this study, we present the cases of 19 consecutive pediatric immunocompromised patients who underwent ECMO for acute respiratory failure. The mortality rate in our cohort reached 73.7%, which aligns with findings in both adult and pediatric patients, where mortality rates around 60-70% have been reported (8, 9, 11, 27). Conversely, the ELSO database indicates a lower overall mortality rate of around 40% for pediatric respiratory ECMO runs during the same period as our study, which corresponds with the overall mortality rate observed at our institution (5, 10).

A monocentric retrospective study conducted in France, which included 111 children treated with ECMO, revealed significantly lower 6-month survival rates (42% vs. 63%) for immunocompromised patients compared to non-immunocompromised patients (7). No significant differences in baseline laboratory or ventilator data were observed between these two groups.

We could not identify prospective studies comparing pediatric immunocompromised ECMO survivors to non-survivors. Nevertheless, a retrospective analysis by Gow et al. using data from the ELSO registry focused on immunocompromised children with malignancies and reported a 35% survival rate to hospital discharge (13). Patients undergoing HCT prior to ECMO therapy were excluded from the analysis. Non-survivors had lower PaO2 and higher OI levels before ECMO therapy. In our patient cohort deceased patients had higher mean- and inspiratory ventilation pressures as well as higher OIs and higher pCO2 levels before ECMO initiation. The differences between the groups were rather small and not statistically significant. In contrast to Gow et al. we found non-survivors to have significantly higher CRP values before initiation of ECMO. It is worth noting that systemic infection with elevated CRP values may pose a risk factor for mortality, particularly in immunocompromised patients undergoing ECMO. Prior studies have demonstrated that immunocompromised children or those who have undergone HCT and develop severe sepsis or septic shock face a significantly higher risk of PICU mortality (28). Furthermore, pre-existing and acquired infections are associated with failure to wean from ECMO (29, 30). In our small cohort no single parameter at baseline was significantly associated with weaning failure from ECMO. Notably, CRP levels - which were significantly correlated with mortality at hospital discharge - were higher (median 87, IQR 157 vs. 193.5, IQR 207.8) in patients who failed to wean from ECMO compared to successfully weaned patients, though the difference did not reach statistical significance.

Consistent with the findings of Gow et al., non-survivors experienced more bleeding complications, as defined by ELSO criteria: a need for more than 20 ml/kg of packed red blood cell transfusion per day or the requirement for surgical or endoscopic intervention (13). However, this was not associated with baseline platelet levels, which were higher in non-survivors. During the ECMO course, platelet counts were maintained above an age-dependent threshold. Bleeding complications are known to be common in pediatric ECMO patients—particularly in immunocompromised individuals—and have been associated with increased morbidity and mortality (7, 9, 31). Bleeding complications may be attributed not only to ECMO and the anticoagulation required to maintain circuit patency, but also to the severity of the underlying illness, including coagulopathy, mucosal vulnerability, and organ dysfunction (e.g. liver failure). In our study the most frequently affected bleeding sites were the oropharynx and the cannulation site. Furthermore, the total number of complications was higher in non-survivors in our study, consistent with reports from the ELSO registry indicating a correlation between complications during the ECMO run and higher mortality rates (5).

Unfortunately, all four patients in our cohort who underwent ECMO therapy after HCT could not be weaned from ECMO. Children after HCT are known to face particularly high risks of poor outcomes, with reported survival rates below 30% (6, 15, 32, 33). Some studies, however, have reported improving PICU and ECMO survival rates in HCT patients over the last two decades (16, 33–35). However, all studies are of retrospective nature, have a small simple size and the improvement is from 10-20% survival 20 years ago to about 30% in the last decade. This is in contrast with our also small and retrospective cohort. The four HCT patients in our cohort were admitted between 2010 and 2019 and were placed on ECMO between 8 and 134 days after HCT. Due to reported high mortality risk and center experience children who received HCT are reluctantly placed on ECMO at our institution. Nevertheless, two recently published consensus statements on ECMO in children receiving HCT strongly recommend considering ECMO in children with non-malignant diseases or with low-risk malignancies and a reasonable expectation of disease-free survival, provided that the critical illness is expected to resolve within a reasonable time frame (18, 20). The authors emphasize the complexities in evaluating ECMO candidacy in pediatric HCT patients, noting the need for comprehensive consideration of factors like disease type, current critical illness, organ reserve, and complications. The complexity of the disease course and significant co-morbidities in high-risk patients like those post-HCT makes predicting outcomes particularly challenging. This further complicates the decision-making process for ECMO candidacy. Acknowledging the improvements over the last decade, ECMO might be a viable option for patients following HCT. However, the diverse spectrum of underlying diseases, patient variability, and the lack of definitive baseline parameters for outcome prediction necessitate a cautious, multidisciplinary, and individualized decision-making process.

All four patients in our cohort who had an underlying primary immunodeficiency (PID) died and three out of four failed to be weaned from ECMO. The severe course, due to the opportunistic infection, was exacerbated in patients 18 and 19 by immune deficiency-associated hyperinflammation. Both patients exhibited a comparatively high inflammatory response at the time of ECMO initiation. Zabrocki et al. reported an odds ratio of 2.35 for mortality in patients with PID requiring ECMO compared to patients without PID (12). A recently published report from the ELSO registry on children with PID who underwent ECMO therapy between 1993 and 2018 showed a survival-to-discharge rate of 45.2% which is higher than overall survival rates reported in the mixed cohort of patients with primary and secondary immunodeficiencies (36). Interestingly neither pre-ECMO infection nor infectious complication during ECMO were associated with non-survival. Moreover, no other reported pre-ECMO baseline parameter was significantly correlated with mortality. However, the occurrence of complications during the ECMO run was significantly associated with mortality.

Our study included 9 patients diagnosed with cancer. Among the cancer patients who did not undergo HCT, 67% (4 out of 6) survived to discharge and were still alive six months after ECMO therapy. Recent meta-analyses on pediatric cancer patients report a 28% PICU mortality rate and a 55% mortality rate in those who received ECMO (37, 38). Therefore, as in our cohort, mortality for pediatric cancer patients appears to be better compared to other immunocompromised patients but worse than for non-immunocompromised patients. However, it is important to note, that estimated mortality rates alone should not be the sole criteria for ECMO allocation to individual immunocompromised patients. Despite the high mortality rate of 74% in our cohort, the five survivors are still alive and experience reasonable health-related quality of life. Four out of five patients have good neurologic outcomes. One patient has a pre-existing severe autism spectrum disorder, the same patient is also suffering from secondary sclerosing cholangitis.

The five survivors did not significantly differ from the non-survivors in any of the baseline parameters except for CRP levels. Although not statistically significant, patients who died showed a trend towards higher vasopressor usage and creatinine levels. Aligned with previously published data the P-PREP score failed to distinguish survivors from non-survivors (32). The P-PREP score was developed and validated to predict mortality in pediatric patients requiring ECMO for respiratory failure (25). It incorporates ventilator and blood gas parameters, which in our cohort did not differ significantly. Furthermore, patients with oncologic diagnoses are categorized into higher-risk groups within the score, although this group showed comparatively favorable outcomes in our study. In contrast, patients with poor outcomes in our cohort—such as those with primary immunodeficiencies—are not specifically accounted for in the score’s risk stratification.

The pSOFA score has been developed to discriminate in-hospital mortality in septic children and has not yet been validated for its use in ECMO (24). A higher SOFA score has been shown to be a risk factor for mortality in adult patients requiring ECMO therapy for cardiac failure as well as respiratory failure due to COVID-19 infection (29, 39). The design of the pSOFA score—which was developed to quantify sequential organ failure in sepsis rather than severe respiratory failure in the complex context of immunodeficiency—may explain its lack of discrimination between survivors and non-survivors in our study. Consequently, there is no established guideline or parameter in the literature to guide ECMO allocation for immunocompromised pediatric patients. Therefore, a case-by-case decision is necessary. For some patients, ECMO could serve as a bridge to responding to the underlying disease treatment, enabling therapy for patients with a reasonable long-term prognosis.

This study presents several noteworthy limitations. Firstly, it is based on a relatively small and heterogeneous group of patients, potentially restricting the generalizability of our findings. The limited sample size might have reduced our ability to detect statistically significant differences or associations. Secondly, as a single-center study, there is a possibility that the results may not fully represent a broader population. Furthermore, the inclusion of patients with various indications for ECMO support introduces variability in patient characteristics and outcomes. The 17-year timespan of our study likely encompasses changes in ECMO technology, patient management, and center-specific experience. Additionally, we were unable to gather long-term follow-up data, such as information on functional, psychological, or social outcomes for both patients and their families. This kind of data could have offered valuable insights into the extended effects of ECMO therapy. In summary, due to the wide spectrum of diseases and the limited number of patients included in the study, drawing further definitive conclusions is not feasible. These limitations should be taken into consideration when interpreting the study’s results and implications discussed in the corresponding section.

Other relationships that might be perceived by the academic community as representing a potential conflict of interest must be disclosed. If no such relationship exists, authors will be asked to confirm the following statement:

## Data Availability

The original contributions presented in the study are included in the article/supplementary material. Further inquiries can be directed to the corresponding author.
